# Work-related physical activity and psychological distress among women in different occupations: a cross-sectional study

**DOI:** 10.1186/s12889-020-09112-7

**Published:** 2020-06-26

**Authors:** Rhiannon Lee White, Jason Bennie, Gavin Abbott, Megan Teychenne

**Affiliations:** 1 School of Health Sciences, Western Sydney University, Locked Bag 1797, Penrith, NSW 2751 Australia; 2 Physically Active Lifestyles Research Group (USQ-PALs), Institute for Resilient Regions, University of Southern Queensland, Springfield, Central QLD 4300 Australia; 3 Institute for Physical Activity and Nutrition (IPAN), School of Exercise and Nutrition Sciences, Deakin University, Geelong, Australia

**Keywords:** Exercise, Mental health, Occupations, Physical activity, Psychological distress, Work, Tradesperson

## Abstract

**Background:**

Recent evidence suggests that work-related physical activity may not have the same mental health benefits as leisure-time physical activity. Further, work-related physical activity is likely to include a variety of different behaviours for people with different occupations. As such, the aim of this study was to determine if occupation type moderated the association between work-related physical activity and psychological distress.

**Methods:**

A randomly selected sample of 1080 women from Melbourne, Australia completed the International Physical Activity Questionnaire (IPAQ) and General Health Questionnaire (GHQ-30), and reported their current occupation.

**Results:**

Linear regression analyses indicated that occupation significantly moderated the association between work-related walking and psychological distress (F [8, 55] = 2.26, *p* = .036). Given evidence of moderation, we fitted linear regression models to test the associations between work-related physical activity and psychological distress for three separate groups; professionals, sales and services workers, and tradespersons. Female tradespersons who engaged in a low (B = − 3.81, *p* = .006) or high amount of work-related walking (B = − 3.23, *p* = .029), had significantly lower psychological distress symptoms than those who engaged in no work-related walking. There were no significant associations between work-related physical activity of any intensity and psychological distress for professionals, or sales and service workers.

**Conclusions:**

Given the relationship does not exist across all occupations, work-related physical activity should not be promoted above and beyond leisure-time physical activity. However, walking at work may be important in reducing psychological distress for some people and should therefore, not be discounted.

## Background

Mental health disorders contribute a substantial proportion to the global burden of disease, and account for 10.4% of the total all-cause disability adjusted life years and an even larger 18.9% of years lived with a disability [1]. Despite increased efforts to understand and prevent mental health disorders, and promote mental health, the gap in life expectancy between the general population and those with a mental health disorder continues to widen [2]. As such, the World Health Organization declared the need for collaborative public health approaches to reduce the pervasive and costly effects of mental health disorders [3]. Abundant evidence shows that physical activity is associated with a reduced risk of mental ill-health, including depression, anxiety, and psychological distress [4]. However, despite ample evidence supporting a relationship between physical activity and mental health, the strength of the relationship varies considerably across studies. This inconsistency needs to be understood so that physical activity can be optimally used to promote mental health and reduce mental ill-health.

Studies show that the relationship between physical activity and mental health varies between different physical activity domains (i.e., areas of life in which physical activity can occur), with meta-analytic evidence demonstrating an inverse association between leisure-time physical activity and mental ill-health only [5]. That meta-analysis also demonstrated that work-related physical activity may in fact be associated with an increased likelihood of depression, anxiety, and psychological distress [5]. However, large heterogeneity existed between the results of individual studies. Additionally, research now shows that higher amounts of work-related physical activity are associated with poor physical health and an increased risk of early mortality [6]. As such, work-related physical activity might not have the same benefits to health and wellbeing as physical activity in other domains, particularly leisure-time.

Despite evidence suggesting that work-related physical activity may not be as beneficial as leisure-time physical activity, and may potentially be detrimental [5, 6], global [7] and national physical activity guidelines [8, 9] encourage adults to be active during any life domain, including leisure-time physical activity, transportation, occupational physical activity, and household chores. Evidence suggests that leisure-time physical activity only accounts for a small portion of adults’ weekly physical activity, with occupational physical activity being the largest contributor [10]. Also, adults with lower levels of education, who often have poorer mental health, engage in more work-related physical activity, compared to adults with higher levels of education [11]. Moreover, approximately half of all people who report no leisure-time physical activity, report 60 min or more of work-related physical activity per day [12]. This suggests that many people may complete the recommended amount of physical activity per week without completing any leisure-time specific physical activity and therefore, may receive no mental health benefit. Further, a recent cohort study showed that compensation occurs when adults transitioned from a sedentary occupation to an active occupation, as participants reduced their physical activity during leisure-time to account for the increased activity at work [13]. While this compensation may result in the same amount of weekly physical activity, it may be associated with poorer mental health if the same mental health benefits are not derived from work-related physical activity, when compared to leisure-time physical activity. However, given the limited studies investigating the association between work-related physical activity and mental health, further evidence is needed. This is especially true for female populations given work-related physical activity has been more frequently examined among male populations. Further, evidence suggests that women report higher levels of psychological distress [14], and typically engage in lower levels of leisure-time physical activity [15] compared to men. Despite engaging in lower levels of leisure-time physical activity [15], data shows that 40% of males and 38% of females participate in strenuous work-related physical activity most or all of the time [11]. Given women’s participation in paid employment has increased steadily since the 1960’s [16, 17], and their employment is as likely to include physical activity, work-related physical activity needs to be better understood among women in terms of psychological distress and wellbeing.

While measuring domain-specific physical activity helps to distinguish different types of physical activity behaviour (e.g., work and leisure), and take context into account, work-related physical activity is still likely to include within it a large variety of different behaviours and movements. For example, an office worker who is mainly seated during desk-based work activities may engage in light walking throughout the day, while typical work-related physical activity for those working in a construction site could include frequent bouts of low to moderate intensity movement, including walking, standing, and lifting. Not only is the behaviour itself likely to be physically different, but, the purpose of the activity and the context surrounding the behaviour is likely to vary also. As such, it seems plausible that the relationship between work-related physical activity and mental health may vary between different occupations. However, to our knowledge, no study has taken into account occupation type, when examining the relationship between work-related physical activity and mental health related outcomes. Therefore, the purpose of this study was to examine the relationships between work-related physical activity and psychological distress among women, and determine if occupation type moderated these relationships.

## Methods

### Participants

Participants were recruited from 45 neighbourhoods (suburbs) in Melbourne, Australia as part of a larger study [18, 19]. The 45 suburbs were selected based on the Australian Bureau of Statistics’ Socioeconomic Index for Areas (SEIFA) which reflects the level of socio-economic advantage and disadvantage of each suburb based on education, occupation, income, household status, and access to resources [20]. A total of 15 suburbs of low-SEIFA were randomly selected, 15 of mid-SEIFA, and 15 of high-SEIFA. Women living in those suburbs between 18 and 65 years were then randomly selected from the electoral roll and 4800 women were sent a questionnaire and invited to participate. The study was approved by the Deakin University Human Research Committee and a total of 1554 women returned the questionnaire and provided consent to participate (32.38% response rate). We excluded 389 participants as they were full-time students or unemployed at the time of data collection, and a further 85 who did not report psychological distress, leaving 1080 participants. There were no significant differences between those who were excluded due to not responding to specific questionnaire items and those included in the analysis in terms of self-reported health (*t* = − 1.68, *p* = .09), BMI (*t* = .72, *p* = .48), or socioeconomic advantage (*t* = −.15, *p* = .88).

### Measures

#### Work-related physical activity

Participants self-reported their work-related physical activity by completing the International Physical Activity Questionnaire – Long Form (IPAQ-L). The IPAQ-L involves seven-day recall of physical activity across four domains (leisure-time, work, transport, and household physical activity). Participants reported the number of days (frequency), and hours and minutes on each day (duration), in each physical activity domain across the past week. Using a standardised protocol [21], total duration of work-related physical activity was calculated by multiplying the frequency and duration within this domain only. This process was completed for each intensity of physical activity to calculate walking, moderate, and vigorous work-related physical activity variables. Total work-related physical activity was then calculated by adding all three intensities together. All four variables had a sizable proportion of zeroes and were consequently recoded into categorical variables based on tertile splits [22]. The IPAQ-L has been shown to have acceptable one-week test-retest reliability (Spearman’s rho = 0.80) and moderate criterion validity (median rho = 0.30) using accelerometery as the standard [21].

#### Psychological distress

We used the 30-item version of the General Health Questionnaire (GHQ-30) to assess participants’ level of psychological distress [23]. The GHQ-30 is a widely used questionnaire that has been extensively validated in adult populations [24] and asks participants if they recently (in the last couple of weeks) experienced a range of psychiatric symptoms. Participants respond to each symptom by selecting the appropriate option indicating better/healthier than normal (scored 0), the same as usual (scored 0), worse/more than usual (scored 1), or much worse/more than usual (scored 1). The GHQ-30 total score ranging from 0 to 30 is commonly used as a continuous variable [25, 26] and has been highly correlated with the Present State Examination index [27]. Alternatively, a threshold total score of > 4 indicates participants at risk of developing psychiatric disorders [28]. As such, we used the validated cut-off score to categorise participants as at-risk or not at-risk in descriptive analyses but, used the total score as a continuous variable for all regression analyses.

#### Occupation category

Based on classification similar to the International Standard Classification of Occupations (2008), participants reported their main occupation as manager or administrator, professional, associate professional, clerical, sales, or service worker, tradesperson, production or transport worker, or labourer. We grouped similar occupations together to create three occupational categories to test for moderation: 1) managers, administrators, and professionals, 2) clerical, sales, and service workers, and 3) tradespersons, transport workers, and labourers. For brevity, these three groups of participants will be referred to as Job Category 1: ‘Professionals’, Job Category 2: ‘Sales and Service’, and Job Category 3: ‘Trades’.

#### Potential confounders

As sociodemographic and lifestyle factors are likely to influence physical activity [15] and psychological distress [29–31], we adjusted inferential analyses for age, education, self-reported height and weight-derived Body Mass Index (BMI), self-reported presence of a physical illness, injury, or disability; and leisure-time physical activity.

### Data analysis

All data analyses were conducted in STATA 14 (StataCorp, TX). Descriptive statistics were used to report participant characteristics (mean, standard deviation, frequencies) for the entire sample, followed by descriptive statistics for each of the three subsamples (i.e., professionals, sales and service, and trades). To test overall associations between work-related physical activity and psychological distress, linear regression analyses were conducted using the total sample, where the GHQ-30 total score (psychological distress) was the dependent variable and the work-related physical activity measures (in separate models) were independent variables. To examine whether the effect of work-related physical activity on psychological distress differed across occupation categories, we fitted our linear regression models with a main effect of occupation category and its interaction with work-related physical activity. Where there was evidence of a moderating effect of occupation category on the relationship between work-related physical activity on depressive symptoms (*p* < .05 for interaction effect), we conducted linear regression analyses stratified by occupation category to examine associations between work-related physical activity and psychological distress for professionals, sales and service, and trades separately. We fitted all regression models with cluster-robust standard errors to account for clustering within suburbs, and excluded participants with missing total (16.20%), walking (19.35%), moderate (17.04%), or vigorous (17.22%) physical activity data in the regression model with that corresponding dependent variable.

## Results

Participants (*n* = 1080) were 18 to 65 year old females (*M* = 41.24, *SD* = 11.69) and were mostly born in Australia (75.9%). The majority of participants were married (66.2%) and nearly half had a university degree (43.6%). Just over half were within a healthy weight range (57.0%), and nearly one-third (29.2%) of participants were at risk of psychological distress (Table 1), which is similar to previous findings among Australian women [32]. Participants’ self-rated health was also similar to results from a previous mail-out survey study conducted in Australia with a much higher 60% response rate [33]. Approximately one-third of participants resided in a neighbourhood of high-SEIFA (34.35%), while slightly more were from a mid-SEIFA neighbourhood (39.38%) and slightly less from a low-SEIFA neighbourhood (26.28%). Participants with trade-related occupations reported more work-related physical activity per week (*M* = 20.17 h) than professionals (*M* = 6.96 h) or sales and service workers (*M* = 9.04 h). Table 1 shows physical activity levels of participants across light, moderate, and vigorous categories, and full participant characteristics are included in Supplementary Material.

**Table 1:** Physical activity levels and psychological distress across different job categories

	**Total Sample (*N*** **= 1080)*N*(%)**	**Professionals (*N*** **= 597)*N*(%)**	**Sales and Service (*N*** **= 349)*N*(%)**	**Trades (*N*** **= 134)*N*(%)**
***Work-related physical activity***				
**Total (hrs/week)**				
Low (< 0.28 h p/week)	302 (32.4)	194 (36.5)	101 (34.2)	7 (6.7)
Medium (0.28–7.33 h p/week)	305 (32.8)	190 (35.8)	97 (32.9)	18 (17.1)
High (> 7.33 h p/week)	324 (34.8)	147 (27.7)	97 (32.9)	80 (76.2)
**Walking (hrs/week)**				
No walking	461 (41.1)	238 (43.5)	129 (41.9)	28 (26.2)
Low (0.1–3.5 h p/week)	235 (29.1)	166 (30.3)	91 (29.5)	23 (21.5)
High (> 3.5 h p/week)	260 (29.8)	143 (26.1)	88 (28.6)	56 (52.3)
**Moderate (hrs/week)**				
No moderate PA	461 (48.2)	292 (54.2)	154 (50.2)	15 (13.6)
Low (0.1–2.67 h p/week)	235 (24.6)	132 (24.5)	78 (25.4)	25 (22.7)
High (> 2.67 h p/week)	260 (27.2)	115 (21.3)	75 (24.4)	70 (63.6)
** Vigorous (hrs/week)**				
No vigorous PA	623 (64.3)	389 (70.9)	196 (63.4)	38 (34.2)
Low (0.1–3 h p/week)	178 (18.4)	101 (18.4)	60 (19.4)	17 (15.3)
High (> 3 h p/week)	168 (17.3)	59 (10.7)	53 (17.2)	56 (50.5)
***At risk of psychological distress***				
Yes (> 4)	315 (29.2)	156 (26.1)	118 (33.8)	41 (30.6)
No (≤4)	765 (70.8)	441 (73.9)	231 (66.2)	93 (69.4)

*PA* physical activity

### Work-related physical activity and psychological distress

No physical activity variables (walking, moderate, vigorous, or total) were significantly associated with psychological distress in the whole sample (Table 2). However, interaction analyses revealed that job category significantly moderated the association between work-related walking and psychological distress (F [8, 55] = 2.26, *p* = .036). Job category did not significantly moderate the association between psychological distress and moderate (F [8, 55] = 0.43, *p* = .898), vigorous (F [8, 55] = 0.68, *p* = .709), or total (F [8, 55] = 1.00, *p* = .447) work-related physical activity.

**Table 2:** Associations between work-related physical activity and psychological distress

	**B**	**95% CI**	***p***
***Total***			
Medium (0.28–7.33 h p/week) vs. Low (< 0.28 h p/week)	0.05	− 0.67, 0.77	.887
High (> 7.33 h p/week) vs. Low (< 0.28 h p/week)	0.18	− 0.53, 0.88	.620
High (> 7.33 h p/week) vs. Medium (0.28–7.33 h p/week)	0.12	− 0.60, 0.85	.732
***Walking***			
Low walking (0.1–3.5 h p/week) vs. No walking	−0.20	− 0.88, 0.48	.561
High (> 3.5 h p/week) vs. No walking	−0.27	− 1.00, 0.47	.471
High (> 3.5 h p/week) vs. Low walking (0.1–3.5 h p/week)	− 0.07	− 0.79, 0.65	.849
***Moderate***			
Low PA (0.1–2.67 h p/week) vs. No moderate PA	0.15	−0.83, 1.12	.764
High PA (> 2.67 h p/week) vs. No moderate PA	−0.03	− 0.81, 0.76	.948
High PA (> 2.67 h p/week) vs. Low PA (0.1–2.67 h p/week)	−0.17	− 1.25, 0.90	.750
***Vigorous***			
Low PA (0.1–3 h p/week) vs. No vigorous PA	0.18	−0.79, 1.15	.709
High PA (> 3 h p/week) vs. No vigorous PA	0.20	−0.73, 1.13	.667
High PA (> 3 h p/week) vs. Low PA (0.1–3 h p/week)	0.02	− 1.17, 1.15	.973

Separate models were tested for each work-related physical activity measure (i.e., walking, moderate, vigorous, total). For rows one and two in each model, the reference category is the lowest PA category (i.e., no PA or low PA). For row three, the highest category is compared to the middle PA category. All models were adjusted for neighbourhood clustering as well as for age, BMI, education status, physical illness, and leisure-time physical activity. *PA* physical activity

After adjustment for potential confounders, those in trade-related occupations engaging in a high volume (> 3.5 h per week) of walking at work had an estimated 3.23 points lower (*p* = .029) psychological distress score compared to those doing no work-related walking (Table 3). Similarly, those engaging in a lower amount (0.1–3.5 h per week) of walking at work had an estimated 3.81 points lower (*p* = .006) psychological distress score compared to those doing no work-related walking. There were no significant associations between work-related walking and psychological distress for professionals or sales and service workers.

**Table 3:** Associations between work-related walking and psychological distress stratified by occupation category

	**B**	**95% CI**	***p***
**Professionals**			
Low walking (0.1–3.5 h p/week) vs. No walking	0.11	−0.65, 0.87	.768
High (> 3.5 h p/week) vs. No walking	0.03	−0.97, 1.02	.954
High (> 3.5 h p/week) vs. Low walking (0.1–3.5 h p/week)	− 0.08	− 1.01, 0.84	.857
**Sales and Service**			
Low walking (0.1–3.5 h p/week) vs. No walking	0.29	−1.20, 1.79	.695
High (> 3.5 h p/week) vs. No walking	0.53	−1.05, 2.12	.500
High (> 3.5 h p/week) vs. Low walking (0.1–3.5 h p/week)	0.24	−1.07, 1.55	.713
**Trades**			
Low walking (0.1–3.5 h p/week) vs. No walking	−3.81	− 6.47, − 1.14	.006
High (> 3.5 h p/week) vs. No walking	−3.23	−6.12, − 0.34	.029
High (> 3.5 h p/week) vs. Low walking (0.1–3.5 h p/week)	0.57	−2.40, 3.55	.698

Separate models were tested for each work-related physical activity measure (i.e., walking, moderate, vigorous, total). For rows one and two in each model, the reference category is the lowest PA category (i.e., no PA or low PA). For row three, the highest category is compared to the middle PA category. All models were adjusted for neighbourhood clustering as well as for age, BMI, education status, physical illness, and leisure-time physical activity

## Discussion

This is the first study to compare different occupations in terms of the relationship between work-related physical activity and psychological distress. The key finding was that among a large sample of women, those with trade, labour, or transport-related occupations, who reported some work-related walking, have significantly lower psychological distress than those who reported no work-related walking. However, in contrast, there were no significant associations between work-related physical activity of any intensity and psychological distress for ‘professionals’ (i.e., professionals, admin workers, or managers); or ‘sales and service workers’ (i.e., clerical, sales, and services workers).

The finding that work-related walking was inversely associated with psychological distress among women working in trade occupations only, could potentially be explained by a sense of satisfaction. As job satisfaction is highly influenced by accomplishments at work [34], it is possible that achieving physical tasks at work provides a sense of accomplishment and satisfaction, which if experienced on a regular basis, is likely to lead to a higher sense of life satisfaction and reduced psychological distress. Among women this may be particularly true as material achievements are more strongly associated with job satisfaction than financial achievements [35]. A recent qualitative study examined people’s perspectives on community gardening, and identified that participants experienced a sense of satisfaction because they contributed to something [36]. Participants also acknowledged that they experienced a great sense of satisfaction by being able to make a difference through their work [36]. While community gardening is a different setting to work, the notion of being satisfied by helping others and contributing to a larger goal that involves interaction and collaboration to build a project, may be similar for people with trade-related occupations whose work involves landscaping or construction, where the work completed leads to benefits for other people. This sense of satisfaction may also evoke similar emotions to experiencing a sense of mastery, which is hypothesised as a mechanism for the mental health benefits of physical activity, as a sense of mastery may benefit mental health due to enhanced feelings of control and success, and increased self-esteem [37]. While accomplishments, a sense of mastery, and job satisfaction are possible across all occupation types, and certainly all employees may experience varying levels of job satisfaction, it is possible that for women with trade-related occupations, the amount of physical activity at work is more closely related, or more integral to, job satisfaction, given these types of jobs are typically more active than other occupations. Among professional or sale related occupations, employees may experience a sense of satisfaction, but physical activity may not be as integral to employee accomplishments.

It is also possible that physical activity within trade-related occupations is more likely to occur outdoors than physical activity within professional or sale and service roles, meaning work-related physical activity for people in job category 3 may be more likely to involve exposure to greenspace. Time spent in greenspace has been associated with lower levels of depression, anxiety, and stress [38, 39] and physical activity outdoors has been associated with greater reductions in depression and anxiety, compared to physical activity indoors [40]. However, we did not assess exposure to greenspace, hence future research should examine whether exposure to greenspace moderates the association between work-related physical activity and mental health related outcomes.

Despite work-related physical activity being associated with reduced psychological distress, this was only the case for walking (i.e., light-intensity physical activity). This finding could be the result of a female only sample, as previous research [41] identified an inverse relationship between light-intensity physical activity and depression in women but an inverse relationship between vigorous-intensity physical activity and depression in men. However, it could also be that moderate and vigorous physical activity at work do not hold the same association with psychological distress as walking. Vigorous physical activity is sometimes associated with feelings of displeasure, particularly among people whose competence or confidence is lacking [42]. Further, the demand-control model [43] proposes that higher occupational demands, either psychological or physical, increase job stress and is inversely associated with psychological wellbeing [44]. Tasks at work that are more physically vigorous may be perceived as more demanding and may therefore lead to more adverse psychological outcomes, when compared to less vigorous tasks. Job control, or autonomy, however, may partially buffer the detrimental effects of job demands on psychological wellbeing [44]. It could be possible that different occupations provide employees with varying levels of job control and a lack of control enables job demands to lead to adverse psychological outcomes within some occupations more than others.

In relation to intensity, previous research also shows that affect was most positive among participants exercising at either lower intensities, or at a self-selected intensity [45]. The option to self-select the intensity of physical activity may not be as possible within the work domain, as it is during leisure-time – where moderate and vigorous physical activity are associated with better psychological outcomes [5, 46]. Specifically within the work domain, the intensity of physical activity may not be a personal choice, and vigorous activities may undermine an employee’s need for autonomy. Autonomy, which is *“the need to self-regulate one’s experiences and actions”* [47], is associated with better mental wellbeing across all life domains, and autonomy support specifically within the workplace is associated with better psychological outcomes [48, 49]. It may be possible that trade-related employees perceive greater autonomy around their involvement in physically active behaviours at work, or perhaps even greater autonomy or control over the intensity at which they complete physically active tasks. Further, autonomy satisfaction is associated with more autonomous motivation towards physical activity and autonomous motivation is consistently associated with psychological wellbeing [48]. Perhaps because physical activity is more integral to trade-related occupations, employees with these types of jobs are more autonomously motivated towards work-related physical activity. In other occupation types, employees may certainly be autonomously motivated towards their job in general, but physical activity at work is seen as more separable to the job itself or their reason for choosing such a job. However, no study has assessed autonomy support, or motivation specifically towards work-related physical activity. Perhaps future research should examine whether these factors moderate the association between work-related physical activity and mental health related outcomes.

While it remains unclear the reasons why work-related physical activity may hold different relationships with psychological distress among different occupations, the current study does suggest that physical activity at work may be differently associated with mental health depending on occupation type. While caution needs to be exercised due to the low response rate and the smaller group of participants working in trade-related occupations, in the future, physical activity guidelines may need to distinguish between leisure-time physical activity and work-related physical activity [5, 6, 50], as it appears that work-related physical activity may be not associated with mental health in the same consistent way as leisure-time physical activity [5, 6]. Also, future research needs to examine why work-related physical activity is beneficial within some occupations and not others.

This study is the first to compare different occupations in terms of the relationship between work-related physical activity and psychological distress, and the first to show that occupation type moderates the association between work-related physical activity and psychological distress. This finding prompts a number of new research questions; nevertheless, a number of limitations should be noted. First, while the results are likely to be generalisable across different ages, since only female participants were included, these results may not be generalisable to males. Therefore, future research should examine the role of occupation type among men. Further, the sample in this study was overrepresented by women with a university degree and by women with professional occupations, when compared to population-based data in Melbourne, Australia [51]. However, because of the three occupational categories, this bias is unlikely to impact the inverse association between work-related physical activity and psychological distress for women with trade-related occupations. We do acknowledge that the findings for professionals and sales and service workers may have been different if the sample was more representative in terms education and occupation type. Nevertheless, the results still present novel findings and suggest future areas of inquiry. Also important to note is that the participants self-reported physical activity which may have introduced social desirability bias, and that there were less participants in occupation category 3 (i.e., tradespersons, transport workers, and labourers) compared to the other two occupation categories. While occupations were categorised with other occupations that were most similar, it is possible that the relationship between physical activity and mental health varies even between similar jobs. In the future, more specific occupation categories should be used. Lastly, a cross-sectional design means that causal relationships could not be determined.

## Conclusion

Physical activity at work does not appear to be consistently associated with psychological distress as the relationship seems to vary between different occupations. As such, a number of mechanisms and potential moderators need to be investigated before we can truly understand how to best promote mental health through workplace physical activity. Nevertheless, walking at work for females with trade, labour, and transport-related occupations may reduce psychological distress symptoms, and therefore, work-related physical activity should not be completely disregarded in terms of its potential for improving mental health.

## Supplementary information

**Additional file 1.** Table 1. Participant Characteristics

## Data Availability

The datasets generated and/or analysed during the current study are not publicly available due to ethical constraints.

## Abbreviations

IPAQ: International Physical Activity Questionnaire
GHQ-30: General Health Questionnaire
SEIFA: Australian Bureau of Statistics’ Socioeconomic Index for Areas
BMI: Body Mass Index
PA: Physical activity
