# Supplementary material for: Work-related physical activity and psychological distress among women in different occupations: a cross-sectional study
Source: BMC Public Health. 2020 Jun 26;20:1007. doi: 10.1186/s12889-020-09112-7 (PMC7318444; doi:10.1186/s12889-020-09112-7)
Supplement: Supplementary file 1 — Additional file 1. Table 1. Participant Characteristics [file 12889_2020_9112_MOESM1_ESM.docx]

**Supplementary Material**

Table 1

Participant Characteristics.

| **Characteristic** | **N** | **%** |
| --- | --- | --- |
| ***Age*** |  |  |
| Under 30 years | 215 | 20.2 |
| 30-39 years | 295 | 27.8 |
| 40-49 years | 279 | 26.3 |
| Over 50 years | 273 | 25.7 |
|  |  |  |
| ***Body Max Index (BMI)*** |  |  |
| Underweight | 29 | 2.9 |
| Healthy weight range | 581 | 57.0 |
| Overweight | 246 | 24.1 |
| Obese | 163 | 16.0 |
|  |  |  |
| ***Country of Birth*** |  |  |
| Australia | 814 | 75.9 |
| UK | 43 | 4.0 |
| Vietnam | 19 | 1.8 |
| Italy | 17 | 1.6 |
| Greece | 17 | 1.6 |
| New Zealand | 7 | 0.7 |
| Other | 155 | 14.5 |
|  |  |  |
| ***Marital Status*** |  |  |
| Married or defacto | 708 | 66.2 |
| Never married | 237 | 22.1 |
| Separated, widowed, or divorced | 125 | 11.7 |
|  |  |  |
| ***Highest qualification*** |  |  |
| No formal qualification or up to year 10 | 173 | 16.2 |
| Year 12, trade apprenticeship, certificate, or diploma | 430 | 40.1 |
| University or higher degree | 466 | 43.6 |
|  |  |  |
| ***Currently has a physical illness/long-term disability*** |  |  |
| Yes | 129 | 12.1 |
| No | 941 | 87.9 |
|  |  |  |
| ***Main Occupation*** |  |  |
| *Job Category 1* |  |  |
| Manager or administrator | 112 | 10.4 |
| Professional | 370 | 34.3 |
| Associate professional | 115 | 10.6 |
| *Job Category 2* |  |  |
| Elementary clerical, sales, or service worker | 53 | 4.9 |
| Intermediate clerical, sales, or service worker | 144 | 13.3 |
| Advanced clerical, sales, or service worker | 152 | 14.1 |
| *Job Category 3* |  |  |
| Tradesperson or related worker | 57 | 5.3 |
| Labourer or related worker | 66 | 6.1 |
| Intermediate production or transport worker | 11 | 1.0 |
|  |  |  |
| ***Depressive symptoms*** |  |  |
| Yes (>4) | 315 | 29.2 |
| No (≤4) | 765 | 70.8 |
